# Microglia activation in the mPFC mediates anxiety‐like behaviors caused by *Staphylococcus aureus* strain USA300

**DOI:** 10.1002/brb3.2715

**Published:** 2022-08-17

**Authors:** Jiao Zou, Weilong Shang, Ling Yang, Tianyao Liu, Lian Wang, Xin Li, Jinghui Zhao, Xiancai Rao, Junwei Gao, Xiaotang Fan

**Affiliations:** ^1^ Department of Military Cognitive Psychology, School of Psychology Third Military Medical University (Army Medical University) Chongqing China; ^2^ Department of Microbiology, College of Basic Medical Sciences Third Military Medical University (Army Medical University), Key Laboratory of Microbial Engineering under the Educational Committee in Chongqing Chongqing China

**Keywords:** anxiety, mPFC, neuroinflammation, Staphylococcus aureus

## Abstract

**Introduction**: *Staphylococcus aureus* (*S. aureus*) is considered as one of the major causative agents of serious hospital‐ and community‐acquired infections. Recent studies have reported that *S. aureus* infection induced neuroinflammation and was linked with some mental disorders. To evaluate the effects of *S. aureus* infection on abnormal behaviors, we conducted the present study.

**Methods**: A *S. aureus* USA300‐infected mouse model was established using bacterial suspension injection into tail vein. A series of behavioral tests were performed after USA300 infection. The expression of cytokines was detected in serum and mPFC. The number and some morphological parameters of microglia were also evaluated by immunofluorescence staining.

**Results**: Anxiety‐like behaviors, instead of locomotor activity impairment or depression‐like behaviors, were observed in mice infected with *S. aureus* USA300 compared with control. *S. aureus* USA300 infection caused overexpression of IL‐6, TNF‐α, and IL‐1β in serum, resulted in microglial over‐activation and excessive release of proinflammatory cytokines in the mPFC. In addition, overexpression of TLR2 accompanied by increased GLS1 and p‐STAT3 was observed in the mPFC of mice infected with *S. aureus* USA300.

**Conclusion**: This study provides evidence that *S. aureus* USA300 infection can lead to neuroinflammation in the mPFC of mice, which may contribute to the development of anxiety.

## 1 INTRODUCTION

Anxiety can be defined as a state of high arousal and negative valence and is characterized by excessive fear and avoidance in the absence of real danger (Craske & Stein, 2016). Anxiety has emerged as a growing public psychological health problem because of its increasing prevalence over the past few decades (Baxter et al., 2013; Yang et al., 2021). Only 30%–50% of people suffering from anxiety achieve remittance despite of the improvement of psychological and pharmacological treatments for anxiety (Farb & Ratner, 2014; Robinson et al., 2019). The exact etiology of anxiety remains poorly understood. Accumulating evidence has suggested microbial infection at various life stages may confer a significant risk of anxiety (Dinan & Cryan, 2017).

*Staphylococcus aureus (S. aureus)* is a Gram‐positive bacterium that causes infection ranging from skin and soft tissue infection to invasive infection, such as sepsis, endocarditis, osteomyelitis, and pneumonia (Tam & Torres, 2019; Turner et al., 2019). Remarkably, the spread of methicillin‐resistant *S. aureus* (MRSA) strains has seriously reduced antibiotic treatment options and brought a global medical concern. In a clinical trial, increased anxiety was noted in children with atopic dermatitis, which is usually associated with *S. aureus* infection (Huang et al., 2021). A recent study has reported that *S. aureus* could cause a series of anxiety‐like behaviors in brain abscess mice, and these abnormal behaviors were improved by the treatment of bactericidal agent ciprofloxacin, sensitive to the *S. aureus* strain (Dey & Bishayi, 2021). However, the potential role of *S. aureus* infection in the development of anxiety‐like behaviors is still unclear.

A growing stream of studies have suggested that *S. aureus*‐induced peripheral inflammatory or neuroinflammation is involved in the pathogenesis of mental disorders (Balczon et al., 2019; Mahanti et al., 2015). *S. aureus* could engage TLR2 to recognize bacterial lipoproteins and induce inflammatory cytokine production in the central nervous system (CNS) to trigger and maintain the neuroinflammatory process (de Morais et al., 2021). A recent study has found that the activation of microglia and upregulation of TLR2 triggered anxiety‐like behaviors in mice (Chen et al., 2021). In addition, clinical and basic studies have proven that microglial activation as well as proinflammatory cytokines, such as TNF‐α and IL‐6, arising from both peripheral and CNS induced by infection or stress response was involved in the development of anxiety‐like behaviors (Ishikawa & Furuyashiki, 2022). Therefore, *S. aureus*‐induced neuroinflammation may play a critical role in anxiety.

The medial prefrontal cortex (mPFC) has been posited to serve a variety of social, affective, and cognitive functions (Lieberman et al., 2019) and is also extremely vulnerable to inflammatory injury. It was reported that the modulation of mPFC was closely related to psychiatric disorders and abnormal behaviors (Adams et al., 2022; Isserles et al., 2021). An animal study has shown that low‐intensity focused ultrasound stimulation could inhibit social avoidance behavior by inhibiting activation of the inflammatory response, increasing neuronal excitation, and protecting the integrity of the neuronal structure in the mPFC (Wang et al., 2022). In a chronic social defeat stress depression model, obvious microglial activation and excess release of proinflammatory cytokines were found in the mPFC of mice with depressive and anxiety‐like behaviors (Dang et al., 2022). The mPFC may be an important brain area mediating anxiety.

In our study, we evaluated the causal relationship between *S. aureus* USA300‐induced neuroinflammation in the mPFC and anxiety‐like behaviors. The results indicated that the upregulation of GLS1 as well as the NF‐κB/STAT3 signaling pathway in the mPFC drive mount neuroinflammation, which is involved in the development of anxiety‐like behaviors caused by USA300 infection. This study provides a new idea for the mechanism and treatment of anxiety.

## 2 MATERIALS AND METHODS

### 2.1 Mice

Male BALB/C mice (6–8 weeks old and weighing 20–24 g) were purchased from Hunan Saike Jingda Experimental Animal Co. Ltd. All mice were housed on a 12/12 light/dark cycle and fed a standard diet with tap water. All testing procedures were performed in a room to minimize any stress response potentially induced by novel environmental cues. All experimental procedures were approved by the Third Military Medical University and performed according to the Guidelines of Laboratory Animal Care and Use.

### 2.2 Bacterial strains and growth conditions

*S. aureus* USA300 strain (GenBank accession no. CP000255.1) was grown with shaking (200 rpm) in brain heart infusion (BHI) medium at 37°C.

### 2.3 Body weight and survival rate monitoring

Male BALB/c mice were randomly divided into the following two groups: control and USA300. Mice were injected with 100 μl saline or USA300 suspension (1 × 10^5^ CFU and 1 × 10^7^ CFU) via tail vein. The body weights and survival rates of mice in different groups were recorded for 8 days.

### 2.4 Behavior tests

All behavioral experiments were performed during the light phase.

#### 2.4.1 Open field test

General exploratory locomotion was assessed in a square gray Plexiglas arena (40 cm × 40 cm × 30 cm). Mice were placed in the center of the apparatus and allowed to move around freely for 30 min. The movements of mice were recorded for 30 min using a video camera secured to the top of the apparatus and analyzed using Ethovision XT 11.0 (Noldus). The test apparatus was cleaned with 70% ethanol between each subject test session (Cai et al., 2017).

#### 2.4.2 Light‐dark transitions

The light‐dark transitions test was used to evaluate the anxiety levels of mice according to previous procedures (Flannery et al., 2015). Mice were placed into the “light” side (∼400 lx) and allowed to move freely for 10 min. Time spent in the dark side and the total number of transitions were automatically recorded by Ethovision XT 11.0 (Noldus).

#### 2.4.3 Elevated plus‐maze

The elevated plus‐maze (EPM) was structured with two open arms and two closed arms (30 cm × 6 cm ×15 cm) extending from a central area (6 cm × 6 cm). Initially, mice were placed in the center area facing an open arm. During the 10 min session, the percentage of time spent in the open arms and the percentage of open arm entries were analyzed. The maze was thoroughly cleaned using 70% ethanol between each test session.

#### 2.4.4 Sucrose preference test (SPT)

Mice were given two bottles in the home cage from which they could drink freely. During the first day, the two bottles contained water and allowed habituation to the bottles. On the following day, one bottle was replaced with a 1% sucrose solution. Mice were then allowed to drink from the bottles over two days, and consumption of each bottle was recorded daily. The bottles were switched daily to ameliorate side bias. Fluid intake was measured afterwards by weighing the drinking bottles. The sucrose preference was calculated from the amount of sucrose solution consumed, expressed as a percentage of the total amount of liquid drunk: sucrose preference = (sucrose intake (g)) / (sucrose intake (g) + water intake (g)) 100% (Williams et al., 2020).

#### 2.4.5 Tail suspension test (TST)

Mice were suspended by a hook 50 cm above the floor using adhesive tape. The hook was placed ∼1 cm from the tip of the tail. The activities of mice were recorded for 6 min. The immobility duration was analyzed during the last 4 min.

#### 2.4.6 Forced swimming test (FST)

A glass cylinder (height: 20 cm, diameter: 10 cm) was filled with water up to a level of 10 cm. The temperature was maintained at 23 ± 2°C. Mice were placed in the middle of the given cylinder and allowed to swim for 6 min. The immobility durations of mice during the last 4 min of the total of 6 min were evaluated by two independent operators.

#### 2.4.7 New object recognition (NOR)

The new object recognition task was conducted in a square gray Plexiglas arena (40 cm × 40 cm × 30 cm) located in a sound attenuated room. Briefly, after a 10 min habituation period, two identical cylinders (A) were introduced in the diagonal corners of the arena. First, mice were placed in the box and allowed to explore freely the objects for 10 min. Second, one of the cylinders was replaced with a new cuboid object (B). The time for exploring freely was 10 min, which was the same as the first trial. Only the second trial was evaluated. An exploratory preference index defined as the ratio of the time spent exploring the cuboid object (B) to the total time spent exploring both objects (A and B) was calculated. Individual movement tracks were analyzed with Ethovision XT 11.0 (Noldus).

#### 2.4.8 Nest building task

Mice were individually housed in a clean cage for 24 h. The nesting material consisting of nearly 2.5 g/5 cm^2^ of square compressed cotton (Ancare, USA) were placed in the middle of the cage. The percentage of the shredded nestlet was recorded after 24 h.

### 2.5 Real‐time PCR

The mPFC tissues were collected, and the mRNA levels of TLR2, NF‐κB, IL‐6, TNF‐α, IL‐4, and IL‐10 in the mPFC were determined by real‐time PCR. Total RNA was extracted from the mPFC using Trizol (Invitrogen, Carlsbad, CA, USA) according to the manufacturer's instructions, and this step was followed by reverse transcription. Quantitative PCR amplification was performed in triplicate using the SYBR Green kit (Takara Company, Japan) with the following program: 1 cycle of 95°C for 30 s, 40 cycles of 95°C for 5 s and 60°C for 30 s. The primer sequences used are listed in Table S1. All data were normalized against the housekeeping control GAPDH expression using the Thermal Cycler Dice Real Time system (TaKaRa Company, Japan).

### 2.6 Cytokine ELISA

Serum IL‐6, TNF‐α, and IL‐1β concentrations were detected by sandwich enzyme‐linked immunosorbent assay (ELISA) and calculated based on the standard curve. Serum cytokine levels were expressed in pg/ml, following manufacturer's protocol. The minimum detectable dose for IL‐6, TNF‐α, and IL‐1β was typically 1.6 pg/ml, 1.88 pg/ml, and 2.31 pg/ml, respectively.

### 2.7 Immunofluorescence

According to our previous method (Fu et al., 2018), mice were anesthetized and transcardially perfused with saline followed by 4% paraformaldehyde (PFA). Subsequently, the brains were postfixed in 30% sucrose solution with 4% PFA. Serial coronal brain sections (30‐μm‐thick) were collected on a cryostat and preserved in a cryoprotectant at −20°C. The sections were incubated with the following primary antibodies (rabbit anti‐ionized calcium binding adapter molecule 1, Iba1, 1:1000, Wako) in 1% bovine serum albumin (BSA) overnight at 4°C, and 1% BSA replaced the primary antibody for the negative control. The next day, the sections were washed and incubated with Cy3‐conjugated (1:500, 2 h; Jackson ImmunoResearch, West Grove, PA, USA) secondary antibodies. Stained specimens were observed and captured using a Zeiss Axivert microscope (Oberkochen, Germany) equipped with a ZeissAxioCam digital color camera connected to the Zeiss AxioVision 3.0 system.

Microglia morphology was quantified according to a previous study (He et al., 2022). Eight‐bit 30 μm z‐stack images of Iba1^+^ cells were obtained with no more than a 2 μm interval between planes. Images were converted to binary by using Image J; Soma size, branch numbers, and branch length of microglia were measured using Image J with the plugin AnalyzeSkeleton.

### 2.8 Western blot

The protein of mouse mPFC tissues were extracted, and protein concentration was measured using a bicinchoninic acid kit (Beyotime Institute of Biotechnology, Shanghai, China). Extracted protein was mixed with 5× loading buffer, electrophoresed in 10% sodium dodecyl sulfate polyacrylamide gel, and transferred onto a polyvinylidene difluoride membrane. The membranes were immersed in 5% skim milk for 2 h at room temperature. Then, the membranes were incubated with the primary antibodies at 4°C overnight. The primary antibodies in our present study were as follows: anti‐GAPDH (1:1000, ZSGB), anti‐Iba1 (1:1000, Abcam), anti‐TLR2 (1:1000, Abcam), anti‐NF‐κB (1:1000, CST), anti‐IL‐6 (1:1000, Abcam), anti‐TNF‐α (1:1000, Abcam), anti‐p‐STAT3 (1:1000, Abcam), anti‐STAT3 (1:1000, Abcam), and anti‐GLS1 (1:1000, CST). The next day, the membranes were washed and incubated with the antirabbit or antimouse secondary antibodies for 1 h at room temperature. The specific protein bands were visualized in membranes by chemiluminescence method, and the optical density of protein bands were analyzed using Bio‐Rad Image‐Lab 6.0 software.

### 2.9 Statistical analyses

All the data were presented as the mean ± SEM and analyzed by SPSS 24.0 software (IBM Corporation, Armonk, NY, USA). Repeated‐measures ANOVA were employed to analyze the subjecting data from the total distance and center time in the OFT and body weight monitoring. Two groups comparison were performed with independent *t*‐tests. The Welch's correction was employed when the variance between the two groups was significantly different, Mann–Whitney *U* test were used when normality of samples was failed according to our previous study (Li et al., 2021). When the *p* value was less than .05, it was considered statistically significantly different in our study.

### 2.10 Ethics approval and informed consent

All experimental procedures were approved by Third Military Medical University and were performed according to the guidelines of laboratory animal care and use.

## 3 RESULTS

### 3.1 Effects of USA300 infection on the body weight and survival rate of mice

The body weights of mice were monitored for 8 days. There was no apparent loss of body weight infected with USA300 (1 × 10^5^ CFU), instead of USA300 (1 × 10^7^ CFU), compared with control (Figure S1a). The survival rate of mice was also monitored for 8 days. All the mice infected with USA300 (1 × 10^5^ CFU) or saline survived. However, the survival rate of mice infected with USA300 (1 × 10^7^ CFU) was obviously decreased (Figure S1b). These results suggested that 1 × 10^5^ CFU, instead of 1 × 10^7^ CFU, USA300 infection could not result in death in mice. Therefore, the dose of 1×10^5^ CFU USA300 was selected to perform the next behavior tests.

### 3.2 Anxiety‐like behaviors were induced in mice infected with *S. aureus* USA300

**FIGURE 1:**
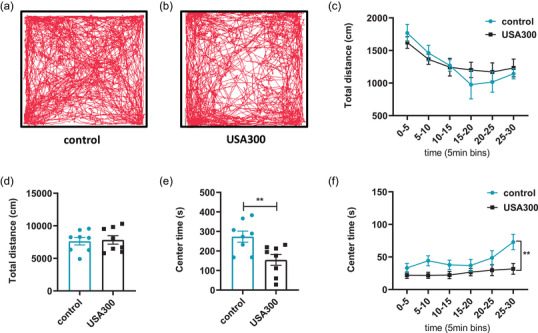
Effects of USA300 on mice in the open field test. (a, b) Representative locomotion tracks traveled over the 30‐min period by the experimental mice in the two groups. (c, d) Mice injected with USA300 exhibited no significant reductions in locomotor activity. (e) Mice injected with USA300 spent less time in the center area. (f) Center time is shown in 5 min time bins. Data are represented as mean ± SEM (*n* = 8 for each group). **p < .01

To explore whether acute *S. aureus* USA300 infection could induce anxiety‐like behaviors, male mice aged 6–8 weeks underwent the open field test, elevated plus‐maze, and light‐dark transition test after tail vein injection. No remarkable difference in the total distance traveled over the total 30‐min period among the USA300 infection and control groups, which indicated that locomotor activity was not affected by *S. aureus* USA300 infection (Figure 1a, b, and d, *p* > .05). The distance traveled in each 5‐min segment decreased with time as the mice gradually acclimatized to the arena (Figure 1c). Mice with a higher degree of anxiety tend to spend more time in the peripheral area of the open‐field arena after adapting to the environment. In the open field test, mice infected with USA300 spent less time in the center area compared to the saline‐treated mice (Figure 1e and f, *p* < .01).

**FIGURE 2:**
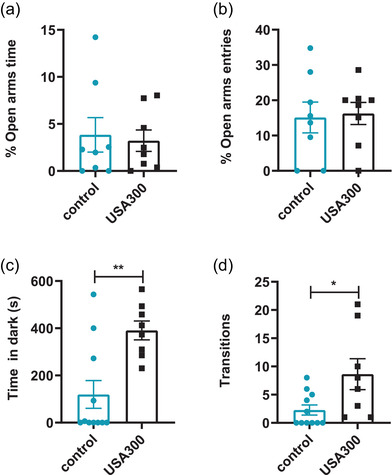
Effects of USA300 on mice in the elevated plus‐maze and the light–dark transition test. (a) Mice injected with USA300 did not alter the percent of the time in open arms. (b) No significant alterations were found in the percentage of entries in the open arms (*n* = 8 for each group). (c) Mice injected with USA300 spent more time in the dark side of the chamber. (d) More transitions were found in mice injected with USA300 (*n* = 11 for control, *n* = 8 for USA300). Data are represented as mean ± SEM. *p < .05, **p < .01

In the EPM test, USA300 infection had no significant effect on the percentage of time in the open arm or the percentage of entries into open arm, compared to the control group (Figure 2a and b, *p* > .05). In the light‐dark transition test, mice infected with USA300 spent more time in the dark box compared to the control group (Figure 2c, *p* < .01). Total transitions of mice infected with USA300 were significantly higher compared to the control (Figure 2d, *p* < .05). Collectively, these results suggested *S. aureus* USA300 infection could induce anxiety‐like behaviors in BALB/C mice.

#### 3.2.1 Effects of *S. aureus* USA300 infection on short‐term memory and depression‐like behaviors

**FIGURE 3:**
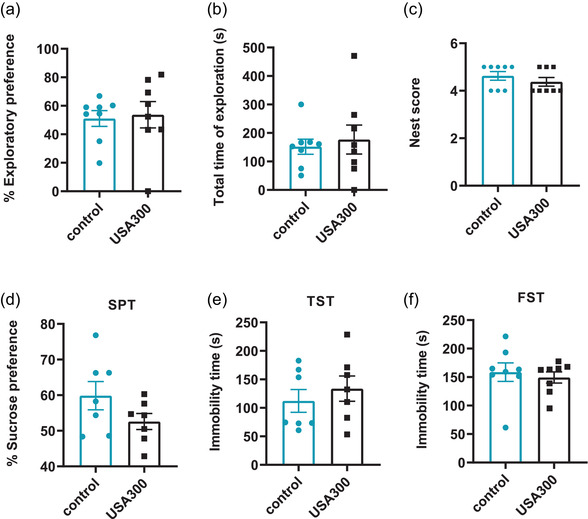
Effects of USA300 infection on short‐term memory, cognitive and depression‐like behaviors in mice. (a) USA300 did not affect the exploration preference of mice. (b) There was no significant difference in the total time of exploration among the two groups (*n* = 8 for each group). (c) There was no significant difference in nesting scores among the two groups (*n* = 8 for each group). (d) Mice in different groups exhibited similar sucrose preference in the sucrose preference test (*n* = 7 for each group). (e) Similar immobility time of mice were found in different groups in the TST (*n* = 7 for each group). (f) Similar immobility time of mice were found in different groups in the FST (*n* = 8 for each group). Data are presented as mean ± SEM

The novel object recognition test was conducted to determine whether *S. aureus* USA300 infection resulted in apparent impairments in the short‐term memories of mice. There was no significant difference in the exploratory preference index between the two groups (Figure 3a, *p* > .05). Moreover, mice in the two groups exhibited similar total exploratory time (Figure 3b, *p* > .05). The nest building task was performed to evaluate the initiative of mice. There was no significant difference in the nesting scores of the two groups (Figure 3c, *p* > .05), suggesting that this non‐learned innate behavior was not impaired by *S. aureus* USA300 infection.

Anxiety and depression are usually regulated by overlapping pathways and occur together in many patients. The effects of *S. aureus* USA300 on depressive‐like behaviors in mice were also measured in this study. The SPT was selected to examine the anhedonia phenotype, which is a core symptom of depression‐like behavior. *S. aureus* USA300 infection did not cause obvious difference in sucrose consumption (Figure 3d, *p* > .05). The tail suspension test and forced swimming test were used to further assess whether *S. aureus* USA300 infection could result in depression‐like behaviors in mice. Increased immobility is a usual measure of behavioral despair. No significantly increased immobility was found in mice infected with *S. aureus* USA300, when compared to the control group in the TST (Figure 3e, *p* > .05) or the FST (Figure 3f, *p* > .05). These results verified that *S. aureus* USA300 infection did not affect the short‐term memory, cognitive or depression‐like behaviors of mice.

### 3.3 *S. aureus* USA300 infection induced cytokine overproduction in serum

**FIGURE 4:**
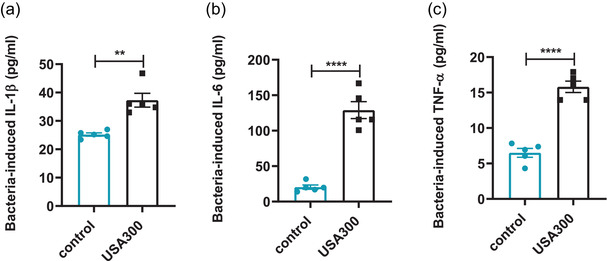
Effects of USA300 infection on the levels of inflammatory cytokines in the serum of mice. (a) Serum level of IL‐1β determined by ELISA. (b) Serum level of IL‐6 determined by ELISA. (c) Serum level of TNF‐α determined by ELISA. Data are presented as mean ± SEM (*n* = 5). **p < .01; ****p ≤ .0001

To determine the host cytokine responses, the production of IL‐1β, IL‐6, and TNF‐α in serum were respectively examined 6 h after *S. aureus* USA300 infection. The levels of serum IL‐1β (Figure 4a, *p* < .01), IL‐6 (Figure 4b, *p* < .0001), and TNF‐α (Figure 4c, *p* < .0001) were significantly elevated in mice infected with *S. aureus* USA300 compared to the control. These results indicated that *S. aureus* USA300 infection induced overproduction of peripheral inflammatory cytokines.

### 3.4 *S. aureus* USA300 infection induced microglia activation and altered proinflammatory cytokine production in the mouse mPFC

**FIGURE 5:**
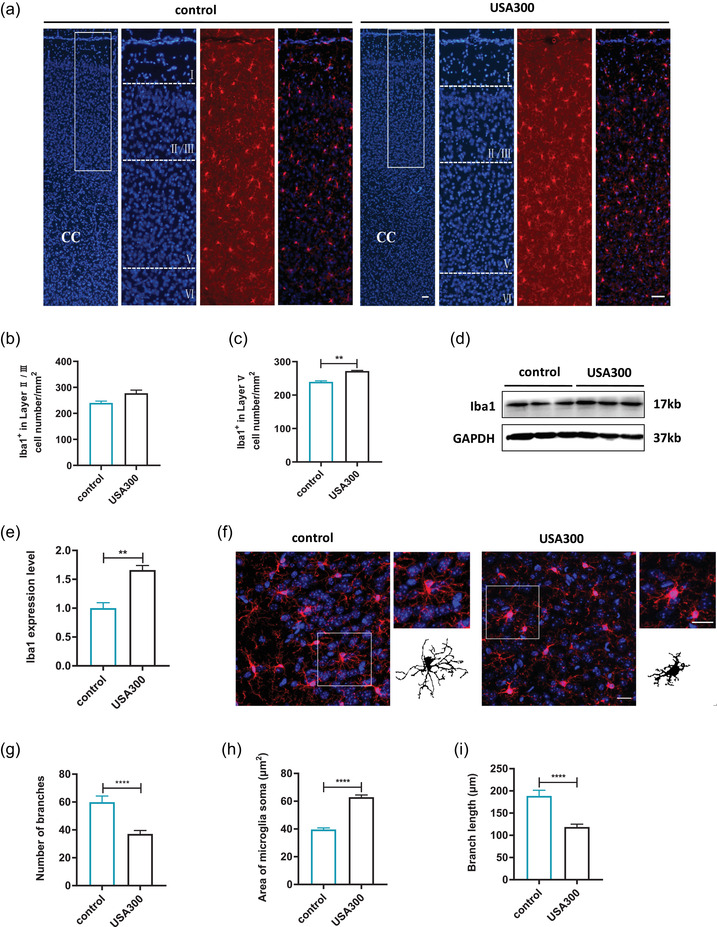
Effects of USA300 infection on the microglia in the mPFC of mice. (a) Microglia labeled by Iba1 in mPFC of the different groups (Iba1, red; DAPI, blue), scale bar = 50 μm. (b) Quantitative analysis of the number of Iba1^+^ cells in layer II/III of mPFC. (c) Quantitative analysis of the number of Iba1^+^ cells in layer V of mPFC. (d) Representative immunoblots of Iba1. (e) Relative expression of Iba1 protein in the mPFC of mice. (f) Morphology of microglia under high power microscopic view and the binary transformation of the representative microglia, scale bar = 20 μm. (g) Quantification of the branch numbers of microglia in mPFC. (h) Quantification of the soma size of microglia in mPFC. (i) Quantification of the branch length of microglia in mPFC. Data are presented as mean ± SEM (*n* = 3).  . **p < .01; ****p ≤ .0001

CNS inflammation mainly involves the activation of microglia cells and the release of proinflammatory cytokines. The activation of microglia in the mPFC was traced by Iba‐1 staining. Light microscopy micrographs of HE‐stained tissues exhibited the different layers of mPFC (Figure S2). The number of microglia labeled by Iba1 was significantly increased in layer V (Figure 5c, *p* < .01), instead of layer II/III (Figure 5b, *p* > .05), of the mPFC of mice infected with *S. aureus* USA300 compared to the control.

In line with the immunohistochemical data, Iba1 protein levels in the mPFC were markedly increased in the mice infected with USA300 compared with the control (Figure 5d and e, *p* < .01). As shown in Figure 5f, microglia exhibited ramified morphology with smaller soma and more microglial processes in the mPFC of control, while microglia displayed an amoeboid morphology, with retracted, thickened processes and enlarged soma in the mPFC of mice infected with USA300. Compared to the control group, microglial soma size was significantly larger (Figure 5h, *p* < .0001) and microglial branch number and length was greatly decreased (Figure 5g and i, *p* < .0001) in mice infected with USA300.

**FIGURE 6:**
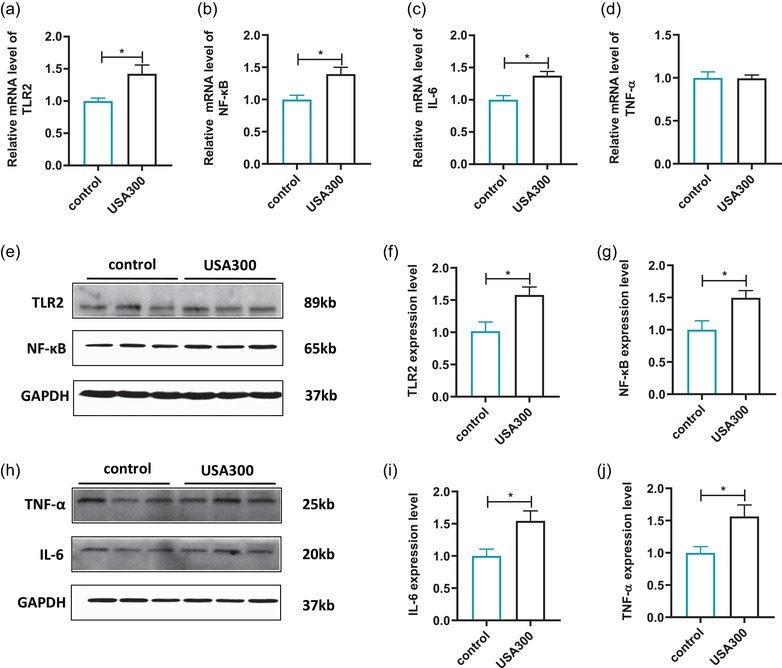
Effects of USA300 infection on inflammatory response in the mPFC of mice. (a) Relative mRNA levels of TLR2 (*n* = 3 for control, *n* = 4 for USA300). (b) Relative mRNA levels of NF‐κB (*n* = 3 for control, *n* = 4 for USA300). (c) Relative mRNA levels of IL‐6 (*n* = 3 for control, *n* = 4 for USA300). (d) Relative mRNA levels of TNF‐α (*n* = 3 for control, *n* = 4 for USA300). (e) Representative immunoblots of TLR2 and NF‐κB. (f) Representative the densitometric analysis of TLR2 (*n* = 3 for each group). (g) Representative the densitometric analysis of NF‐κB (*n* = 3 for each group). (h) Representative immunoblots of IL‐6 and TNF‐α. (i) Representative the densitometric analysis of IL‐6 (*n* = 3 for each group). (j) Representative the densitometric analysis of TNF‐α (*n* = 3 for each group). Data are presented as mean ± SEM. *p < .05

Alterations in the neuroinflammatory cytokines and TLR2/NF‐κB in the mPFC were further analyzed by RT‐qPCR and western blot. The mRNA and protein levels of TLR2 were enhanced in the mPFC of mice infected with *S. aureus* USA300 compared with the control (Figure 6a, e, and f, *p* < .05). In addition, *S. aureus* USA300 infection dramatically upregulated the mRNA and protein levels of NF‐κB (Figure 6b, e, and g, *p* < .05), as well as downstream IL‐6 (Figure 6c, h, and i, *p* < .05). Although there was no significant difference in the mRNA expressions of TNF‐α in the mPFC of mice in the two groups, the protein levels of TNF‐α were significantly increased in the mice infected with *S. aureus* USA300 compared to the control (Figure 6d, *p* > .05; Figure 6h and j, *p* < .05). In addition, the mRNA levels of anti‐inflammatory cytokines IL‐4 and IL‐10 were detected and not statistically changed in the mPFC of mice infected with *S. aureus* USA300 compared to the control (Figure S3a and b, *p* > .05). These results suggested that *S. aureus* USA300 infection induced neuroinflammation in mPFC through TLR2/NF‐κB signaling pathway.

### 3.5 *S. aureus* USA300 induced excessive expression of GLS1 and STAT3 activation in the mPFC

**FIGURE 7:**
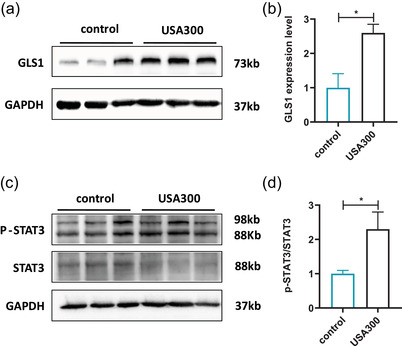
Effects of USA300 infection on the expressions of GLS1 and STAT3/p‐STAT3 in the mPFC of mice. (a). Representative immunoblots of GLS1. (b) Representative the densitometric analysis of GLS1. (c) Representative immunoblots of p‐STAT3 and STAT3. (d) Representative the densitometric analysis of p‐STAT3 and STAT3. Data are represented as mean ± SEM (*n* = 3). *p < .05

Increasing evidence showed that GLS1 plays an important role in neuroinflammation (Ji et al., 2021). In the CNS, GLS is an enzyme that catalyzes the hydrolytic deamidation of glutamine to glutamate and plays an important role in excitotoxic glutamate generation. The expression of GLS1 in the mPFC was detected using western blot. As shown in Figure 7a and b, the levels of GLS1 in the mPFC of *S. aureus* USA300‐infected mice were significantly higher than those of control mice (*p* < .05). Importantly, glutamate derived from glutaminolysis seems to play an essential role in STAT3 activation, which has been increasingly considered to play a critical role in anxiety (Shentu et al., 2021). Western blot was used to further assess the expression of p‐STAT3. Consistently, we found that the p‐STAT3 level was significantly upregulated in *S. aureus* USA300‐infected mice compared to the control group (Figure 7c and d, *p* < .05). These results indicated that the overexpression of GLS1 after *S. aureus* USA300 infection was associated with the activation of the STAT3‐signaling pathway.

## 4 DISCUSSION

In this work, we found that acute *S. aureus* USA300 (1 × 10^5^ CFU) infection resulted in anxiety‐like behaviors. Increased inflammatory cytokines in serum and mPFC were observed in the *S. aureus* USA300‐infected mice accompanied by excessive activation of microglia in the mPFC and upregulation of TLR2. *S. aureus* USA300 infection also upregulated the GLS1 and STAT3‐signaling pathway, implicated with anxiety‐like behaviors.

*S. aureus* is an important Gram‐positive human pathogen that causes serious hospital‐ and community‐acquired infections, including bacterial infections, bacteremia, and sepsis (Vautor et al., 2009). At present, *S. aureus* is one of the most common organisms found in abscess related to traumatic brain injury and neurosurgical procedure (Lu et al., 2002; Patel & Clifford, 2014). Patients with *S. aureus* infection are accompanied by impairments in cognition, memory, sensory processing, and emotional functioning (Iwashyna et al., 2010; Lazosky et al., 2010; Semmler et al., 2013). Mounting clinical evidence has revealed that *S. aureus* infection was closely related to anxiety (Andersson et al., 2016; Mursaloğlu et al., 2021). Dey and Bishayi (2021) have found that 2.0×10^8^ CFU of *S. aureus* (AG‐789) could cause a series of anxiety‐ and depression‐like behaviors in a brain abscess model of Swiss albino mice. In addition, male albino mice were given 5 × 10^6^ CFU of *S. aureus* (ATCC‐6538) intraperitoneal injection and exhibited obvious anxiety‐and depression‐like behaviors (Shal et al., 2019). However, our results indicated that mice infected with 1 × 10^5^ CFU *S. aureus* (USA300) showed obvious anxiety‐like behaviors evaluated by the OFT and light‐dark transitions tests. The difference in behaviors might be due to the concentration and strains of infected *S. aureus*. To further verify that the dose of *S. aureus* is appropriate, we monitored the body weight and survival rate changes after tail vein injection with 100 μl saline or USA300 suspension. 1×10^5^ CFU, instead of 1×10^7^ CFU, *S. aureus* USA300 suspension infection did not result in an apparent loss of body weight or survival rate change compared to the control group.

Bacterial invasion always leads to host inflammation, an adaptive response triggered by noxious stimuli and conditions (Medzhitov, 2008). There is strong evidence that excessive inflammatory response plays a crucial role in the development of anxiety (Duivis et al., 2013; Pitsavos et al., 2006). A recent meta‐analysis has shown that peripheral IL‐6 and TNF‐α were significantly increased in patients with generalized anxiety disorder (Costello et al., 2019). Consistent with this, we demonstrated that the serum levels of IL‐6 and TNF‐α were significantly higher in mice infected with *S. aureus* USA300 compared to the control group. In addition, activation of TLR2 could mediate intracellular signaling in defense against *S. aureus* infection (Barton & Medzhitov, 2003; Esen et al., 2004), trigger TLR2‐MyD88 signaling, and then result in activating NF‐κB signaling via a cascade of intermediary steps. Subsequently, a series of proinflammatory cytokines, such as IL‐6 and TNF‐α, were released (Nguyen & Gotz, 2016). Downregulation of TLR2 could prevent the overexpression of TNF‐α, IL‐1β, and IL‐6 caused by *S. aureus* in mice (He et al., 2021). Some previous studies have pointed out that the imbalances of pro‐ and anti‐inflammatory cytokines were relation with abnormal behaviors (You et al., 2011). IL‐4 and IL‐10 were reported to play important roles in abnormal behaviors (Zhang et al., 2021; Zhang et al., 2020). The mRNA levels of IL‐4 and IL‐10 in the mPFC were detected by real‐time PCR. We found that the levels of IL‐4 and IL‐10 in the mPFC of mice infected with USA300 were not significantly changed compared to the control group. It meant that USA300 infection may not affect the expression of IL‐4 and IL‐10 in the mPFC.

The mPFC is a primary target of stress and connected with many complex behaviors in human and animal studies. The stimulation of extraneous factors could give rise to microglia activation and excessive inflammation in mPFC, which were associated with many psychiatric disorders, such as anxiety and depression (Yang et al., 2021; Yang et al., 2020). Our previous study showed that loss of LXRβ in astrocytes in mPFC leaded to anxiety‐like behaviors in mice (Li et al., 2021). In line with these studies, microglia activation was found in the mPFC after *S. aureus* infection, accompanied by the increase of TLR2/NF‐κB and overexpression of proinflammatory cytokines IL‐6 and TNF‐α in the mPFC. These results indicated that neuroinflammation following *S. aureus* infection in the mPFC was associated with anxiety‐like behaviors. Hippocampus is involved in learning and memory and is also extremely vulnerable to environmental factors. Several studies have suggested that hippocampus may be the other targeted brain region of *S. aureus* infection. Peripheral *S. aureus* infection could promote histone H3 hypoacetylation and decreases tyrosine hydroxylase protein level in the hippocampus and prefrontal cortex of rat (Choudhury et al., 2019). Mice challenged intraperitoneally with *S. aureus* showed increase of anxiety‐like behaviors, which may be mediated by the overexpression of IL‐6 and TNF‐α in prefrontal cortex and hippocampus (Shal et al., 2019). We should pay more attention to the hippocampus after *S. aureus* infection in the following study.

Glutaminase (GLS) mediates the conversion of glutamine to glutamate, supporting most excitatory neurotransmission in the CNS (Kosten et al., 2019). GLS have two isoforms GLS1 and GLS2. GLS1 is the main glutamine metabolic enzyme in the brain rather than GLS2. High levels of GLSl protein were identified in many psychiatric and neurological diseases (Hamed et al., 2018; Huang et al., 2011). Microglial GLS1 deficiency could mitigate neuroinflammation in the lipopolysaccharide (LPS)‐induced depression model (Ji et al., 2021). JHU083, a glutaminase inhibitor, was reported to attenuate behavioral deficits and improve disease outcomes in some neuroinflammation models, such as chronic social defeat stress and Alzheimer's disease (Hollinger et al., 2020; Zhu et al., 2019). In addition, Chen et al. (2012) found that TNF‐α contributed to GLS upregulation and increased glutamate release in Japanese encephalitis virus‐infected microglia. Consistent with these results, we confirmed an increased level of GLS1 in the mPFC following *S. aureus* USA300 infection, suggesting that abnormal GLS1 induced by excessive inflammatory response contributed to anxiety‐like behaviors. Glutaminolysis can regulate the activation of STAT3, and GLS1 inhibition was reported to reduce phosphorylated STAT3 expression (Guo et al., 2016; Xia et al., 2018). Moreover, we found that excessive inflammatory cytokines induced an elevation of STAT3 via enhancing the phosphorylation of the latter in mPFC after *S. aureus* USA300 infection. These results reveal that *S. aureus* USA300 induced excessive expression of GLS1 and STAT3 activation in mPFC.

## 5 CONCLUSIONS

In conclusion, we provided the evidence that *S. aureus* USA300 infection could result in anxiety‐like behaviors, which was involved with neuroinflammation in mPFC. These findings extend our understanding of *S. aureus* infection in the nervous system and may provide novel insights to the model and therapy of anxiety.

## CONFLICT OF INTEREST

The authors confirm that there are no conflicts of interest.

## AUTHOR CONTRIBUTIONS

JZ and WS designed and conducted the experiments, collated and analyzed the data, and wrote the original manuscript. LY and LW contributed to conception and design of the study. XL, TL, and JZ monitored the status of the mice and collected the samples. LY and LW contributed to the collection of data for quantification and statistical analysis. XF, JG, and XR reviewed the experimental plan, provided technical and financial support, and revised the manuscript. All authors contributed to the article and approved the submitted version.

### PEER REVIEW

The peer review history for this article is available at https://publons.com/publon/10.1002/brb3.2715

## Supporting information

FIGURE S1. Effects of USA300 infection on the mouse weight and survival rate of mice
FIGURE S2. Different layers were identified using HE staining in the mPFC of mice
FIGURE S3. Effects of USA300 infection on anti‐inflammatory cytokines response in the mPFC of mice
TABLE S1. Primers used in this study

## Data Availability

The data in this study are available from the corresponding author based on the reasonable request.
