# Supplementary material for: Microglia activation in the mPFC mediates anxiety‐like behaviors caused by Staphylococcus aureus strain USA300
Source: Brain Behav. 2022 Aug 17;12(9):e2715. doi: 10.1002/brb3.2715 (PMC9480961; doi:10.1002/brb3.2715)
Supplement: Supplementary file 1 — FIGURE S1. Effects of USA300 infection on the mouse weight and survival rate of mice FIGURE S2. Different layers were identified using HE staining in the mPFC of mice FIGURE S3. Effects of USA300 infection on anti‐inflammatory cytokines response in the mPFC of mice TABLE S1. Primers used in this study [file BRB3-12-e2715-s001.docx]

**Supplementary Figure 1. Effects of USA300 infection on the mouse weight and survival rate of mice.**

**
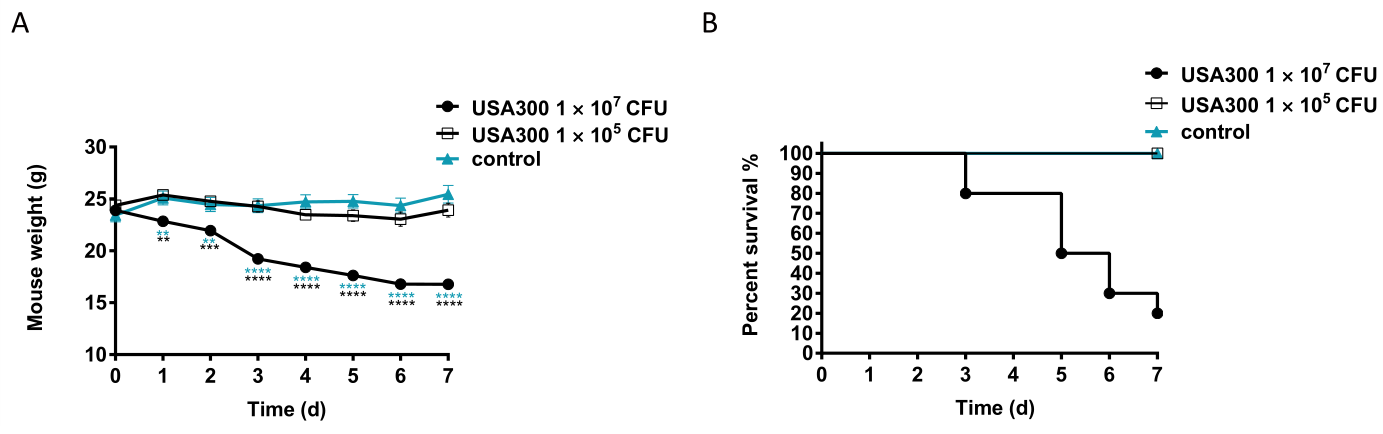
**

(A). The effects of USA300 infection (1×10^5^ CFU and 1×10^7^ CFU) on body weights of mice. (B). The survival rates of mice infected with USA300 (1×10^5^ CFU and 1×10^7^ CFU) and control. Data are presented as mean ± SEM. (n=8 for control, 1×10^5^ CFU, n= 10 for 1×10^7^ CFU). ***P* < 0.01, ****P* ≤ 0.001, *****P* ≤ 0.0001.

**Supplementary Figure 2. Different layers were identified using HE staining in the mPFC of mice.**

**
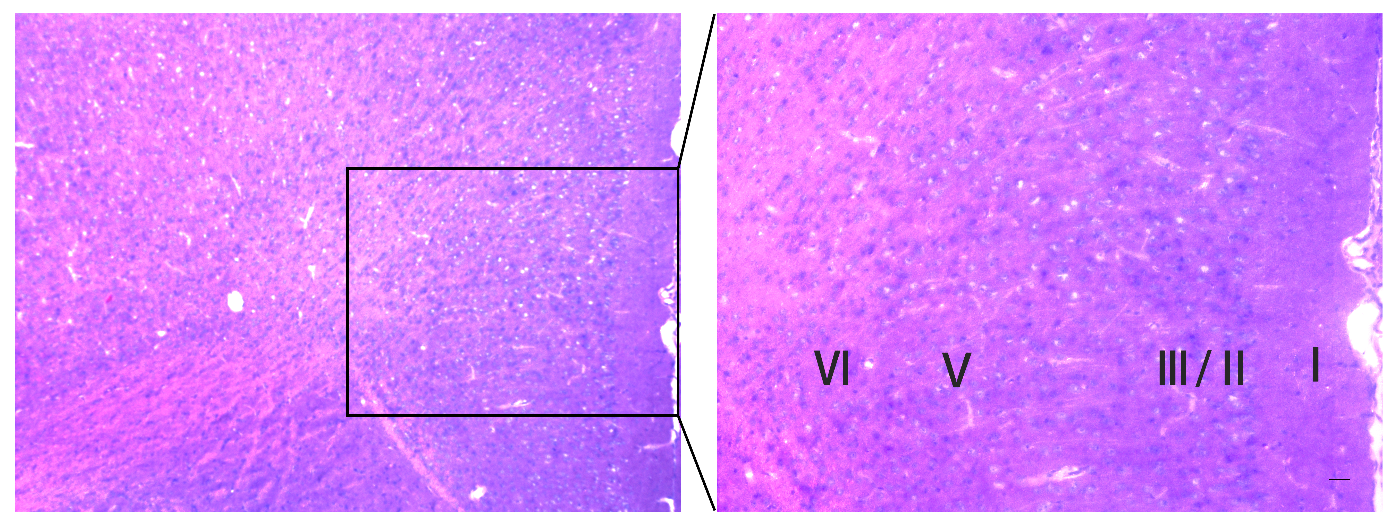
**

Different layers of mPFC were identified using HE stain. scale bar = 50 µm.

**Supplementary Figure 3. Effects of USA300 infection on anti-inflammatory**
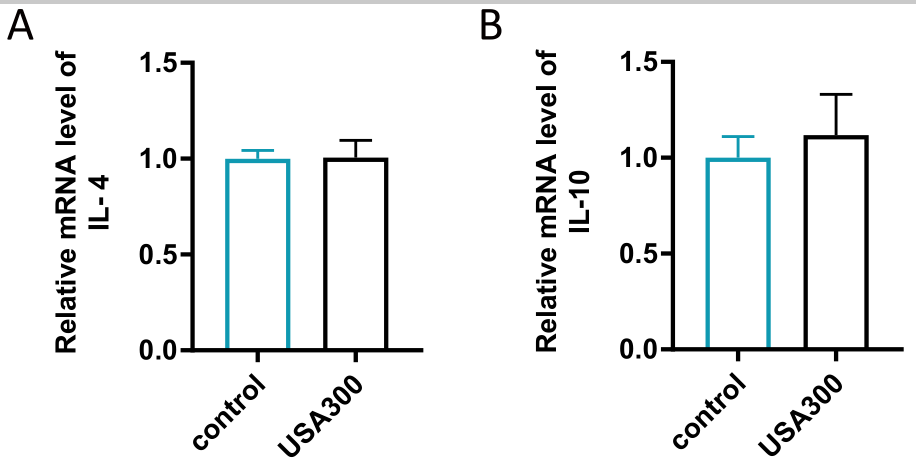
**cytokines response in the mPFC of mice.**

(A). Relative mRNA levels of IL-4; (B). Relative mRNA levels of IL-10. Data are represented as mean ± SEM (n=3 for control, n= 4 for USA300).

**Supplementary Table 1.** Primers used in this study.

| Primers | Sequence (5'-3') | Notes | References |
| --- | --- | --- | --- |
| RT-TLR2-for | gcaaacgctgttctgctcag | RT-qPCR | [1] |
| RT-TLR2-rev | aggcgtctccctctattgtatt |  |  |
| RT-NF-κB-for | atggcagacgatgatccctac | RT-qPCR | [2] |
| RT-NF-κB-rev | cggaatcgaaatcccctctgtt |  |  |
| RT-IL-6-for | ccaagaggtgagtgcttccc | RT-qPCR | [3] |
| RT-IL-6-rev | ctgttgttcagactctctccct |  |  |
| RT-TNF-α-for | gacgtggaactggcagaagag | RT-qPCR | [4] |
| RT-TNF-α-rev | ttggtggtttgtgagtgtgag |  |  |
| RT-IL-4-for  RT-IL-4-rev | ggtctcagcccccaccttgc  ccgtggtgttccttgttgccgt | RT-qPCR | [5] |
| RT-IL-10-for  RT-IL-10-rev | cttactgactggcatgaggatca  gcagctctaggagcatgtgg | RT-qPCR | [6] |
| RT-GAPDH-for | aggtcggtgtgaacggatttg | RT-qPCR | [2] |
| RT-GAPDH-rev | tgtagaccatgtagttgaggtca |  |  |

**References：**

1. Boytard L, Hadi T, Silvestro M, Qu H, Kumpfbeck A, Sleiman R, Fils KH, Alebrahim D, Boccalatte F, Kugler M *et al*: **Lung-derived HMGB1 is detrimental for vascular remodeling of metabolically imbalanced arterial macrophages**. *Nat Commun* 2020, **11**(1):4311.

2. Liu T, Ma Y, Zhang R, Zhong H, Wang L, Zhao J, Yang L, Fan X: **Resveratrol ameliorates estrogen deficiency-induced depression- and anxiety-like behaviors and hippocampal inflammation in mice**. *Psychopharmacology (Berl)* 2019, **236**(4):1385-1399.

3. Hadi T, Boytard L, Silvestro M, Alebrahim D, Jacob S, Feinstein J, Barone K, Spiro W, Hutchison S, Simon R *et al*: **Macrophage-derived netrin-1 promotes abdominal aortic aneurysm formation by activating MMP3 in vascular smooth muscle cells**. *Nat Commun* 2018, **9**(1):5022.

4. Li X, Zhang Q, Shi Q, Liu Y, Zhao K, Shen Q, Shi Y, Liu X, Wang C, Li N *et al*: **Demethylase Kdm6a epigenetically promotes IL-6 and IFN-beta production in macrophages**. *J Autoimmun* 2017, **80**:85-94.

5. You Z, Luo C, Zhang W, Chen Y, He J, Zhao Q, Zuo R, Wu Y: **Pro- and anti-inflammatory cytokines expression in rat's brain and spleen exposed to chronic mild stress: involvement in depression**. *Behav Brain Res* 2011, **225**(1):135-141.

6. Zhang R, Cai Y, Xiao R, Zhong H, Li X, Guo L, Xu H, Fan X: **Human amniotic epithelial cell transplantation promotes neurogenesis and ameliorates social deficits in BTBR mice**. *Stem Cell Res Ther* 2019, **10**(1):153.
